# Clinical and Therapeutic Notes

**Published:** 1887-12-31

**Authors:** C. R. Illingworth


### CLINICAL AND THERAPEUTIC NOTES.

BY C. R. ILLINGWORTH, M.D., M.R.C.S.

PYREXIA AND ANTI-PYRETICS—(*continued.*)

THE mere reduction of temperature never yet cured any disease. To give antipyretics is simply and solely to treat symptoms; and, unless attention be paid to the origin of the disease in prescribing any given drug of this nature, harm may as frequently result as good. From a consideration of the action of the salicylate of soda in reducing the temperature of the body in acute inflammatory affections—and it should here be noted that the affection known as scarlatinal nephritis most assuredly does not come under this category—it is evident that the list of antipyretics may be largely increased. Taking the group of inflammatory diseases, there are several remedies which, like the salicylate of soda, diminish the fibrin of the blood. Amongst these are ammonia, the salicylates of ammonia and potash, benzoate of soda, and the nitrate and acetate of potash. Some exert an action upon the skin, others upon the kidney; but the effect is the same, reduction of the temperature by "liquefacient" action. Again, in the septic group, iron, by increasing the fibrin-forming power of the blood, becomes an antipyretic of a very high order. In the same way, or rather upon the same principle, the biniodide of mercury, by its germicidal action, rids the economy of a source of irritative fever, and may thus be included in the list of antipyretics for the radical or abortive treatment of specific febrile disorders such as scarlet fever, diphtheria, typhoid, etc.

It is thus evident, that the list of pure antipyretics should include those remedies only whose action is exerted upon the nervous system directly, and this can be said to have been conclusively demonstrated in regard to very few of the number of drugs in use for antipyretic purposes. Indeed, if we are to understand that antipyretics are remedies which act only through the nerve centres for the reduction of temperature in disease, it may reasonably be doubted if there are any drugs known to which the term can appropriately be applied, seeing that aconite, antimony, digitalis, gelsemium, ipecacuan, quinine, &c., are antipyretic, mainly, if not altogether, in virtue of their cardiac depressant properties.

Before leaving the subject of antipyretics some of the diseases in which *antipyrin* has been found useful deserve mention, and the theories which have been advanced regarding its action, some consideration. The *Medical Press and Circular* says: "Antipyrin is coming more and more into favour, and will soon supersede quinine. Its manifold properties are being discovered daily." Speaking of its good effects in *migraine*, Dr. Edward Warren Bey, says: "I was recently called to Mrs. F——, fifty-one years of age, who was suffering from this complaint, to which she had been a martyr for several years, never having passed a single day without an attack of greater or lesser intensity. I immediately prescribed antipyrin, giving it in doses of fifteen grains three times a day. I saw her again to day, and learned that the first dose gave marked relief, and that from the second day of the treatment up to the present time she had been entirely free from pain." Dr. Theodore Maxwell in the *Lancet* of the 12th ultimo, also writes: "My attention was first directed to this use of the drug by a paper by Professor Khomyarkoff and Dr. L'voff, which appeared in the *Vrach* of December 19th, 1885; and, as far as I know, the credit of first suggesting antipyrin for the cure of migraine belongs to these Russian physicians. Since that time I have employed it with very great, though not invariable, success. Sometimes the first dose of fifteen grains has acted "like magic" within twenty minutes. In one inveterate case of periodically recurring migraine, where the pain affected a single spot on the right parietal bone, the scalp over which used to become very tender, I was able for many months to give up morphia injections, antipyrin arresting the attacks almost entirely. In this case, however, there have been one or two attacks which no amount of antipyrin would relieve. In other cephalalgias, which cannot be described as migraine, I have found antipyrin useful. Its employment in headaches and neuralgias due to ophthalmic affections was pointed out by Dr. Kalsauroff (*Vrach* No. 7, 1886, and the *Lancet*, April 10th, 1886). In many headaches of nervous, and in some which appeared to be of rheumatic origin, I have found antifebrine or acetanilide, in doses of from five to ten grains, very effective. I have not, however, been able to satisfy myself that it is equal to antipyrin in true migraine. It would be of advantage if it were so, as it is far cheaper than antipyrin."

Dr. M. Byvalkevitch, of the Vilna Military Hospital, prescribes antipyrin in cases of pulmonary hœmorrhage, whether it be due to phthisis, cardiac disease, or traumatic injury. The dose given is five grains, and it is repeated every two or three hours. The hæmoptysis in every case was arrested after the second dose, and in some ergot, ergotin, digitalis, atropine, and Haller's elixir had previously been tried without effect.

In regard to the investigations which have been made upon the alleged nervine antithermic properties of antipyrin, the Swiss correspondent of the *British Medical Journal*, writes: "There exist several distinct regions of the brain, which stand in intimate relation to heat production. The most accessible of these cerebral thermic centres are situated on the medium surface of the striated bodies, and in the subjacent layers down to the base. A puncture of one of these centres is followed by a considerable rise of the systemic temperature, which lasts from twenty-four to forty-eight hours."

Dr. H. Girard has proved that his "artificial fever" is caused solely by lesion of the centre referred to, and that the phenomenon is a true "nervous fever." By experiment he tried to determine how antipyrin acts on the striated thermic centre in healthy rabbits. The results may be summarised as follows: (1) When introduced under the skin in doses of from ·75 to 1·0 gramme, antipyrin invariably lowers the temperature in healthy adult rabbits, sometimes as much as 1·4 C. (2) The drug effectively neutralises excessive heat production caused by the stimulational puncture of the striated thermic centres. (3) In a previously antipyrinised animal the lesion of the median surface of one striated body gives rise to fever as usual, but the latter is always decidedly less intense than in a non-antipyrinised rabbit. The thermo-sedative action of the drug, however, is neither energetic nor prolonged enough to enable it to completely inhibit the phenomena of excitement produced by the lesion of the thermic centres. (4) Antipyrin slows the systemic metabolism (as a diminution in the total quantity of nitrogen in the urine shows), and, like other toxic substances, temporarily induces other changes in the nucleus of the hepatic cells which promote decomposition of such substances, and in this way makes them less injurious to the animal economy. This temporary malnutrition (*dénutrition passagère*) is probably dependent upon an inhibitory action of certain parts of the central nervous system. On the whole, antipyrin must be placed amongst those antithermic remedies which act on the nerve centres, and which may be properly termed "nervine antipyretics." Such is Dr. Girard's argument. It is no proof, however, that, because antipyrin exerts its antipyretic action whilst Dr. Girard is experimenting with the *corpora striata*, that the drug is necessarily operating upon the same centre. It is much more in accordance with the symptoms observed during the administration of the drug, to aver that it acts primarily upon the red blood corpuscles partially to prevent the oxygenation of their hæmoglobin. This action of the drug upon the blood has a rapid effect upon the heart, reducing the strength of the pulsations, and thus upon the respiration, which becomes slower. Drowsiness and diminished tissue change naturally result.

*(To be continued.)*

